# Sequential stable isotope analysis reveals differences in dietary history of three sympatric equid species in the Mongolian Gobi

**DOI:** 10.1111/1365-2664.12825

**Published:** 2016-11-17

**Authors:** Martina Burnik Šturm, Oyunsaikhan Ganbaatar, Christian C. Voigt, Petra Kaczensky

**Affiliations:** ^1^ Research Institute of Wildlife Ecology University of Veterinary Medicine Vienna Austria; ^2^ Great Gobi B Strictly Protected Area Administration Takhin Tal Gobi Altai Province Mongolia; ^3^ Department of Zoology School of Biology and Biotechnology National University of Mongolia Ulan Bator Mongolia; ^4^ Leibniz Institute for Zoo and Wildlife Research Berlin Germany; ^5^ Present address: Norwegian Institute for Nature Research – NINA Trondheim Norway

**Keywords:** Asiatic wild ass, Dzungarian Gobi, *Equus ferus przewalskii*, *Equus hemionus*, feeding ecology, isotope analysis, isotopic dietary niche, pasture competition, Przewalski's horse

## Abstract

1. Competition among sympatric wild herbivores is reduced by different physiological, morphological and behavioural traits resulting in different dietary niches. Wild equids are a rather uniform group of large herbivores which have dramatically declined in numbers and range. Correlative evidence suggests that pasture competition with livestock is one of the key factors for this decline, and the situation may be aggravated in areas where different equid species overlap.
2. The Dzungarian Gobi is currently the only place where two wild equid species coexist and share the range with the domesticated form of a third equid species. In the winter‐cold Gobi desert pasture productivity is low, highly seasonal, and wild equids additionally face increasing livestock densities.
3. We used stable isotope chronologies of tail hairs to draw inferences about multi‐year diet seasonality, isotopic dietary niches and physiological adaptations in the Asiatic wild ass (khulan), re‐introduced Przewalski's horse, and domestic horse in the Mongolian part of the Dzungarian Gobi.
4. Our results showed that even in the arid Gobi, both horse species are predominantly grazers, whereas khulan are highly seasonal, switching from being grazers in summer to mixed feeders in winter. The isotopic dietary niches of the two horse species were almost identical, did not vary with season as in khulan and were narrower than in the latter. Higher δ^15^N values point towards higher water use efficiency in khulan, which may be one reason why they can exploit pastures further away from water.
5. *Synthesis and applications*. The high degree of isotopic dietary niche overlap in the two horse species in the Mongolian Gobi points towards a high potential for pasture competition during the critical nutritional bottleneck in winter and highlights the need to severely restrict grazing of domestic horses on the range of the Przewalski's horses. The evolutionary more distant khulan are less constrained by water and seem more flexible in their choice of diet or less successful in exploiting grass‐dominated habitats in winter due to human presence. Providing additional water sources could increase the competition between khulan and livestock and should, therefore, only be done following careful consideration.

## Abstract 2

The high degree of isotopic dietary niche overlap in the two horse species in the Mongolian Gobi points towards a high potential for pasture competition during the critical nutritional bottleneck in winter and highlights the need to severely restrict grazing of domestic horses on the range of the Przewalski's horses. The evolutionary more distant khulan are less constrained by water and seem more flexible in their choice of diet or less successful in exploiting grass‐dominated habitats in winter due to human presence. Providing additional water sources could increase the competition between khulan and livestock and should, therefore, only be done following careful consideration.

## Introduction

Competition within an assemblage of sympatric herbivores is reduced by different physiological, morphological and behavioural traits resulting in different food requirements in respect to digestibility, amount and access (Gwynne & Bell 1968; Arsenault & Owen‐Smith 2002). Wild equids are a rather uniform taxonomic group which share a common body plan, show little variation in life‐history traits and have a digestive system adapted to process large quantities of low‐quality food (Ransom & Kaczensky 2016). Given the equids’ digestive similarities, Janis (1976) speculated that ‘if horses are competing with ruminants in the grazing community, there may be room for large numbers of individuals in a specialized niche at the top end of the fibre tolerance range, but there would be little room for diversification into numerous species within this niche’. However, wild horses (*Equus ferus*), Przewalski's horses (*Equus* (*ferus*) *przewalskii*) and Asiatic wild asses (or khulan, *Equus hemionus*) once roamed the Eurasian steppe well into the Holocene. Although the exact taxonomic status and the ranges of the three species remain somewhat disputed, there is evidence of considerable past range overlap (Heptner, Nasimovich & Bannikov 1966).

Nowadays, the wild horse is extinct but has become widely replaced by its domesticated form, the domestic horse (*Equus caballus*; Warmuth *et al*. 2012). The khulan has undergone a dramatic reduction in range and population size, with the Gobi region of southern Mongolia and northern China constituting a last stronghold, containing >80% of the global population. The Przewalski's horse was driven to extinction in the wild and only survived due to captive breeding efforts. The two key factors for range restriction and population decline in both species were direct persecution and competition with livestock for pasture and water (Kaczensky *et al*. 2015; King *et al*. 2015). Reintroductions since the 1990s have brought the Przewalski's horse back to parts of its native range, and currently, the Dzungarian Gobi in southwestern Mongolia and northern China is the only place where Przewalski's horses and khulan are again sympatric (Kaczensky *et al*. 2016, 2008).

Equids are generally believed to be grazers, but browsing has been documented when grass is scarce or of low quality (Schoenecker *et al*. 2016). The dry, shrub‐dominated Gobi is believed to constitute an edge, rather than prime Przewalski's horse habitat (van Dierendonck & DeVries 1996; Kaczensky *et al*. 2008). Consequently, Przewalski's horses may be susceptible to competition with the more mobile and numerous khulan, although locally (e.g. at water points) the larger Przewalski's horse with its strong social bonds may be dominant over khulan (Zhang *et al*. 2015). The range of both wild equids also overlaps with domestic horses and other livestock, primarily sheep and goats. Although pasture degradation is of national concern and has already been linked to high livestock density in Mongolia (Hilker *et al*. 2014), present national policy still encourages increasing livestock numbers. On a national level, some 400 Przewalski's horses and 42 000 khulan currently exist alongside 52 million heads of livestock, 3 million of which are domestic horses (Kaczensky *et al*. 2015, 2016; see Fig. S1.1, Appendix S1, Supporting Information). Although, correlative evidence suggests that high livestock densities exclude khulan and Przewalski's horses (Harper 1945; Bannikov 1981; Kaczensky *et al*. 2011b), little comparative data on feeding ecology are available to help assess the potential for pasture competition among the most similar species in the wild equid–domestic livestock interface.

Stable isotope analysis of continuously growing and isotopically inert tissue has become a powerful tool to address individual seasonal variation in diet and water use in a range of large herbivores (DeNiro & Epstein 1978; Cerling *et al*. 2006; Lehmann *et al*. 2015). The underlying principle is that animal tissues reflect the isotope values of the forage and water consumed. Carbon (C) isotopes are particularly useful to address feeding ecology, as plants follow two main different photosynthetic pathways (referred to as C_3_ and C_4_), resulting in profoundly and consistently different C isotope values (O'Leary 1981). However, while stable isotope analysis allows differentiating between isotopically contrasted food groups, it does not provide information at the plant species level, and hence, the isotopic dietary niche is not to be confused with the realized dietary niche. In the cold steppes of Central Asia and Mongolia, grasses primarily follow the C_3_ pathway, and most shrubs and semi‐shrubs follow the C_4_ pathway (Pyankov *et al*. 1999; Su *et al*. 2007; Wittmer 2008). Other isotopes can provide additional information, e.g. hydrogen (H) and oxygen isotope ratios allow inferences on water use (Dansgaard 1964; Hobson, Atwell & Wassenaar 1999; Burnik Šturm *et al*. 2015) and nitrogen (N) isotope ratios can provide insight into the physiological status (e.g. starvations; McCue & Pollock 2008) or the water‐use efficiency of an organism (Ambrose & DeNiro 1986). Within this context, the isotopic dietary niche concept has become widely used to characterize diet width (Shipley 1999; Bearhop *et al*. 2004) and the flexibility to switch between different feeding regimes as a consequence of varying ecological conditions (Codron *et al*. 2013).

In this study, we use longitudinally sampled equid tail hair to obtain individual, temporally explicit, multi‐year information of the dietary regime and water‐use efficiency of two wild and one domestic, free‐ranging equid species in the Mongolian Gobi. With horses generally believed to be adapted to mesic and khulan to arid conditions, and assuming that domestic horses profit from the local herding regime, we expected to find that (i) domestic horses include the highest proportion of grass in their diet, khulan the lowest, and Przewalski's horses intermediate levels, (ii) shrub use in all species is highest during winter when forage is most limited, (iii) the dietary isotopic niches of the two horse species are more similar than those of khulan, and more uniform among individuals as khulan have much larger ranges and show little movement coordination among individuals, (iv) physiological adaptations allow for a more efficient water use in khulan as compared with the two horse species. By comparing two sympatric wild equids with the most similar sympatric livestock species, we shed light on the feeding ecology of this little studied herbivore guild and assessed the potential for grazing competition to help inform ongoing conservation efforts for wild equids in the Mongolian Gobi and elsewhere in the region.

## Materials and methods

### Study area

**Figure 1:**
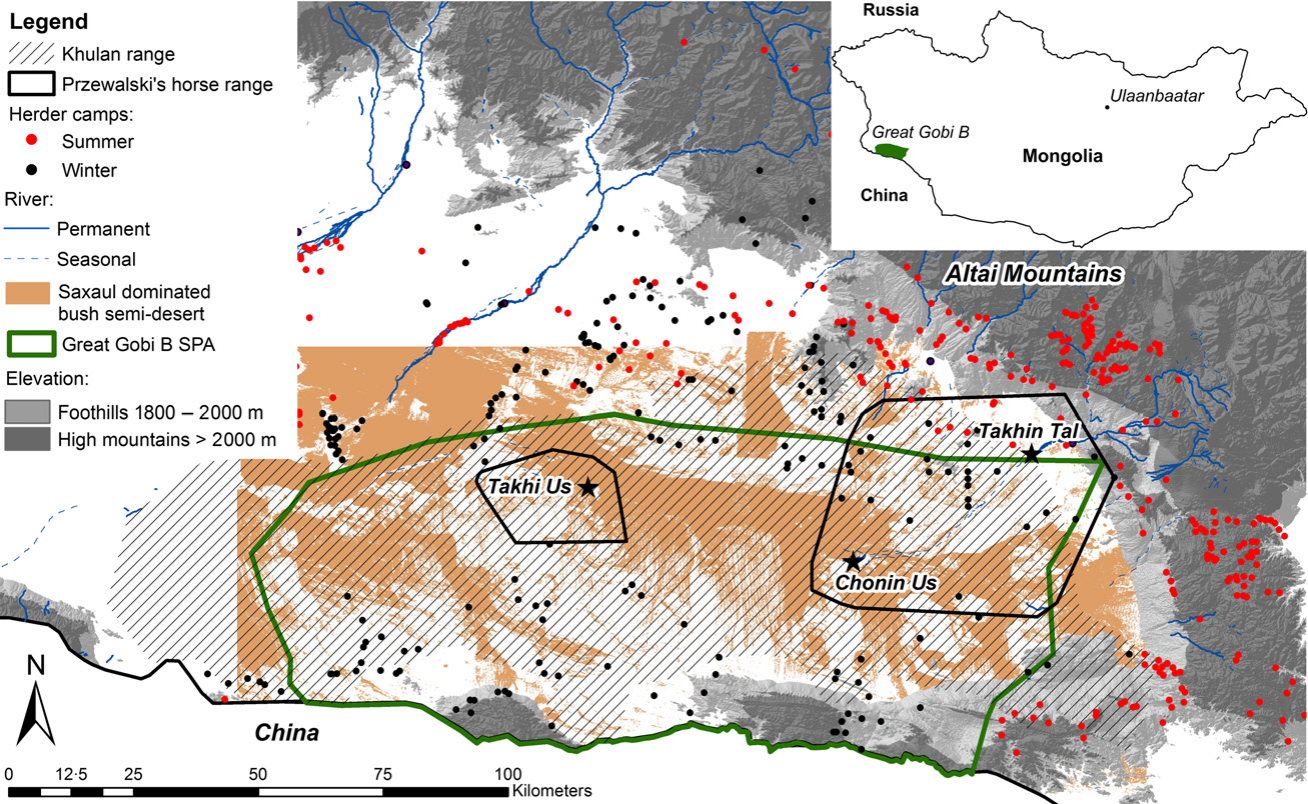
Great Gobi B strictly protected area in the Dzungarian Gobi, southwestern Mongolia.

The Great Gobi B Strictly Protected Area (SPA) stretches over 9000 km² and covers a large part of the Dzungarian Gobi in southwestern Mongolia (Fig. 1; Kaczensky *et al*. 2008, 2011a). Elevations range from 1000 to 2840 m above sea level. The climate is strongly continental with monthly temperatures averaging +17·9 °C in summer (June–August) and −17·9 °C in winter (December–February), with extremes ranging from +35 to −43 °C. Average rainfall is 100 mm with a distinct peak in summer. Average snow cover lasts around 100 days, but is rarely compacted and does not often exceed 20 cm. Open water is rare, particularly in the central and western part of the protected area.

The SPA consists of semi‐arid and arid drylands. The semi‐desert areas are widely dominated by Chenopodiaceae, such as saxaul (*Haloxylon ammodendron*), a large shrub or small tree, and *Anabasis brevifolia*, a semi‐shrub, both of which follow the C_4_ photosynthetic pathway (Pyankov *et al*. 1999; Wen & Zhang 2011). The desert‐steppe areas are dominated by Asteraceae, such as *Artemisia* spec. and *Ajania* spec., and Poaceae like *Stipa* spec., all of which are C_3_ plants (Wittmer 2008; Sun *et al*. 2013). The prevailing plant communities are shrub‐dominated *H. ammodendron* associations (40% coverage) and grass‐dominated *Stipa gobica* and *S. glareosa* associations (35% coverage; Fig. 1; von Wehrden *et al*. 2006).

Herder camps are allowed outside of the 1800 km^2^ core zone at pre‐established locations during winter and are used by about 100 semi‐nomadic herders. Livestock had grown considerably until 2009/2010 when it crashed from some 150 000 to 50 000 (Kaczensky *et al*. 2011a). Over 90% of the livestock are sheep and goats, but even with a share of only about 3% domestic horses still number in the thousands (Fig. S1.2, Appendix S1). Domestic horses are largely free‐ranging and receive no additional fodder, but may occasionally be watered at wells. In summer, human and livestock presence in the park is negligible as herders move to the productive foothills or alpine summer pastures in the Altai Mountains (Kaczensky *et al*. 2008). In winter, herders primarily stay in the grass‐dominated foothills of the SPA's mountains (Fig. 1, Fig. S1.3, Appendix S1).

The khulan population was estimated at 5671 (95% CI = 3611–8907) in 2010 (Ransom *et al*. 2012). The Przewalski's horse population has been reintroduced since 1992 and reached 129 individuals in 2009, but crashed to 49 in the dzud (extreme weather event) winter 2009/2010 (Kaczensky *et al*. 2011a). Przewalski's horses primarily roam over two separated areas around the two reintroduction sites: Takhin Tal in the east (~2000 km^2^) and Takhi Us in the west (~400 km^2^; Fig. 1). As of January 2016, there were 132 Przewalski's horses (O. Ganbaatar, unpublished data).

### Plant and tail hair sample collection

During two sampling expeditions in June/July 2012 and September 2013, we collected 240 samples of 13 C_3_ and two C_4_ plant species throughout the SPA and parts of the Altai Mountains. The sampling was focused on plants known to be frequently consumed by wild equids based on the literature (Bannikov 1981; Sietses *et al*. 2009; Xu *et al*. 2012) and direct observations by SPA staff and local herders. We did not find a significant effect of elevation, longitude or year (data not shown) and subsequently characterize C_3_ and C_4_ plants, respectively, by pooling values over all species and years (see Appendix S2 for further details on plant sampling and plant isotope values).

For each equid species, we plucked several long tail hairs from six adult individuals. As we were interested in species‐specific patterns, we selected for those individuals (i) which allowed for maximum diversity in respect to sex (3 ♂ and 3 ♀) and group composition/membership, (ii) had the longest tail hairs for maximal temporal coverage, and (iii) for which we had additional background information (for details, see Appendix S3).

### Sample preparation and stable isotope analysis

Sample preparation and isotope measurements are described in detail in Appendix S3 and in Burnik Šturm *et al*. (2015). Briefly, in the laboratory, plant samples were re‐dried, homogenized by grinding, weighed (2·0–2·5 mg) and packed into tin cups for C and N stable isotope analysis. Hair samples were cleaned to remove any dirt or fat and cut into 10‐mm segments. Subsamples were then enclosed into tin (for CN) or silver cups (for H) for isotope analysis at the stable isotope facility of the Leibniz Institute for Zoo and Wildlife Research in Berlin. Precision of the measurements was always better than 0·1‰ for δ^13^C and δ^15^N and 1·0‰ for non‐exchangeable δ^2^H based on the repeated analysis of the laboratory standards, calibrated with international standards (for details see Appendix S3).

### Data analysis

For correct temporal assignment of the tail hair increments, we used the high seasonal variation of δ^2^H values in precipitation which results in winter lows and summer highs of δ^2^H values of available water sources which in turn is reflected in the δ^2^H isotope profiles of sequentially cut tails hairs. Briefly, to match the environmental seasonality, we individually adjusted the average species‐specific tail hair growth rate so that the δ^2^H minima are spread apart by 12 months and used the sampling date as explicit end date to create the corresponding time line (for more details, see Burnik Šturm *et al*. 2015).

We converted the isotope ratios of equid hair (δ^15^N_hair_, δ^13^C_hair_) to diet coordinates (δ^15^N_diet_, δ^13^C_diet_) and calculated the dietary components of C_3_ and C_4_ biomass using the dual‐mixing model of Cerling *et al*. (2007). The model takes into account that hair isotope values represent dietary inputs from three pools: the short‐term (with 0·5‐day half‐life, with fraction contributions: 41% for C and 40% for N), intermediate (4‐day half‐life, 15% for C, 12% for N) and long‐term pool (138‐day half‐life, 44% for C, 48% for N). We used the turnover parameters for δ^15^N_diet_ and δ^13^C_diet_ after Ayliffe *et al*. (2004). To calculate the % C_4_ shrubs consumed, we used C_3_ and C_4_ end‐members based on the average δ^13^C values (±1σ) of our 240 plants samples and the diet–hair fractionation factors for horses on a low protein diet: 2·7‰ for C (Ayliffe *et al*. 2004) and 1·9 ‰ for N (Sponheimer *et al*. 2003).

We used one‐way ANOVAs with the Tukey᾽s post hoc HSD to compare the means of isotope tail hair values among species and generalized additive models with integrated smoothness estimation to visually present the species‐specific trends of the δ^13^C_diet_, δ^15^N_diet_ and δ^2^H_hair_ values over time. Models included ‘individual’ as random effects and the AR1 autocorrelation coefficient rho to account for repeated measurements of single hair samples. We visually inspected residuals for normality and independence using histograms and QQ plots. When needed, we transformed data to comply with normality criteria. To derive a standard error that is independent of the random factor, we added a dummy variable. To determine the impact of pasture seasonality on diet, we aligned the diet and isotope profiles with time‐matched 16‐day Normalized Difference Vegetation Index values (Pettorelli *et al*. 2011).

To estimate species‐specific isotopic dietary niche widths and overlaps, we used a Bayesian approach based on bivariate, ellipse‐based metrics (for δ^13^C and δ^15^N values) using SIBER implemented in the R package SIAR (Parnell *et al*. 2010; Jackson *et al*. 2011). Core isotopic niche sizes are expressed as the standard Bayesian ellipse area (SEA_B_) in ‰^2^. We additionally quantified (i) species‐specific isotopic niches for summer, winter and the intermediate periods (spring + autumn) for seasonal comparison among species and (ii) individual isotopic niches for assessing variation within each species (for details, see Appendix S4). All statistical analyses were conducted in R (version 3.1.1).

## Results

### Dietary isotopic baseline

δ^13^C values of plants followed a bimodal distribution based on their photosynthetic pathways (Table S2, Appendix S2). The mean δ^13^C value of C_3_ plants was −25·5 ± 1·3‰ (1σ, *n* = 198, range from −28·9‰ to −22·7‰) and of C_4_ plants was −13·5 ± 0·5 ‰ (1σ, *n* = 42, range from −12·6‰ to 14·5‰). δ^13^C values of individual C_3_ plant species varied up to 5·5‰, as a result of growing in different micro‐environments (Ehleringer & Cooper 1988). Mean δ^15^N values of C_3_ plants had a significantly lower mean value (6·0 ± 2·8‰, 1σ) than C_4_ plants (8·5 ± 2·5‰), but the overall range of C_3_ and C_4_ plants was broad and largely overlapped.

### Mean isotope values of equid tail hair

Mean δ^13^C values did not differ between the two horse species, but were significantly higher in khulan. Mean δ^15^N values were similar between the horse species, but slightly and significantly higher and less variable in khulan. Mean δ^2^H values differed significantly between species: being highest in khulan, intermediate in Przewalski's and lowest in domestic horses (Table 1, Table S3, Appendix S3).

**Table 1:** Mean stable isotope ratios in the tail hair of three equid species, each represented by six individuals

Species	δ^13^C (‰)		δ^15^N (‰)		δ^2^H (‰)	
	Mean ± SD	*N*	Mean ± SD	*N*	Mean ± SD	*N*
Khulan	−21·2 ± 1·7***	275	8·7 ± 1·0***	275	−130 ± 16***	271
Przewalski's horse	−23·4 ± 0·9	308	8·2 ± 1·8***	308	−151 ± 20***	291
Domestic horse	−23·2 ± 0·2	369	8·4 ± 1·5**	369	−161 ± 16***	350

** and *** denote significant differences at *P* < 0·01 and *P* < 0·001, respectively.

### Seasonal isotope patterns

For C, khulan showed a clear and consistent seasonal pattern, indicative of a high proportion of C_4_ plants in the diet (up to 67%) in winter and a low proportion (<10%) in summer. No such pattern was observed in Przewalski's and domestic horses where the proportion of C_4_ diet remained below 20% throughout the year.

For N, no distinct and consistent seasonal trend was evident among individual animals, although the overall model suggested higher values in domestic horses in summer and lower in winter. None of the three species showed consistently elevated δ^15^N_hair_ values in winter, not even Przewalski's horses which all succumbed to the 2019/2010 dzud winter.

**Figure 2:**
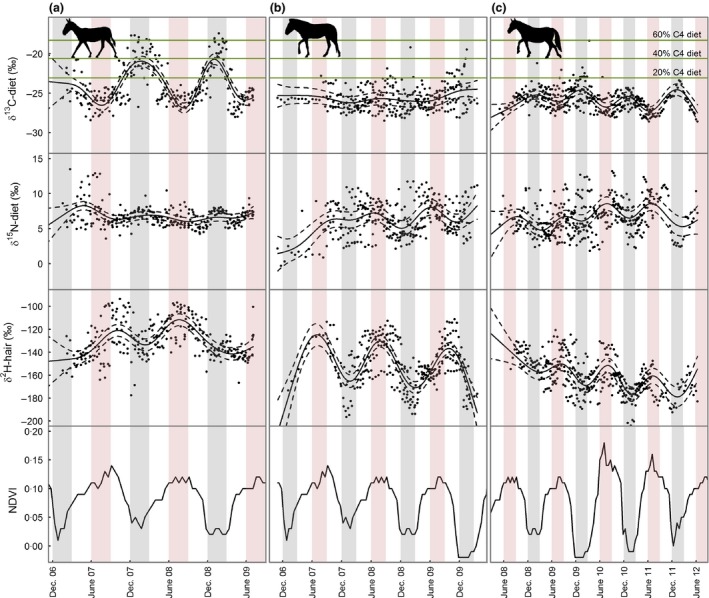
δ^13^C_diet_, δ^15^N_diet_, δ^2^H_hair_ profiles aligned with NDVI in (a) khulan, (b) Przewalski's horses and (c) domestic horses (*n* = 6 per species). Solid lines depict the predicted mean effect for over time and the dotted lines the 95% confidence intervals. Grey shadings depict winter and pink shadings summer.

For H, all three species showed a clear and consistent seasonal pattern with δ^2^H summer highs and δ^2^H winter lows; amplitudes were most distinct in Przewalski's horses (Fig. 2, Appendix S3).

### Isotopic dietary niches

**Figure 3:**
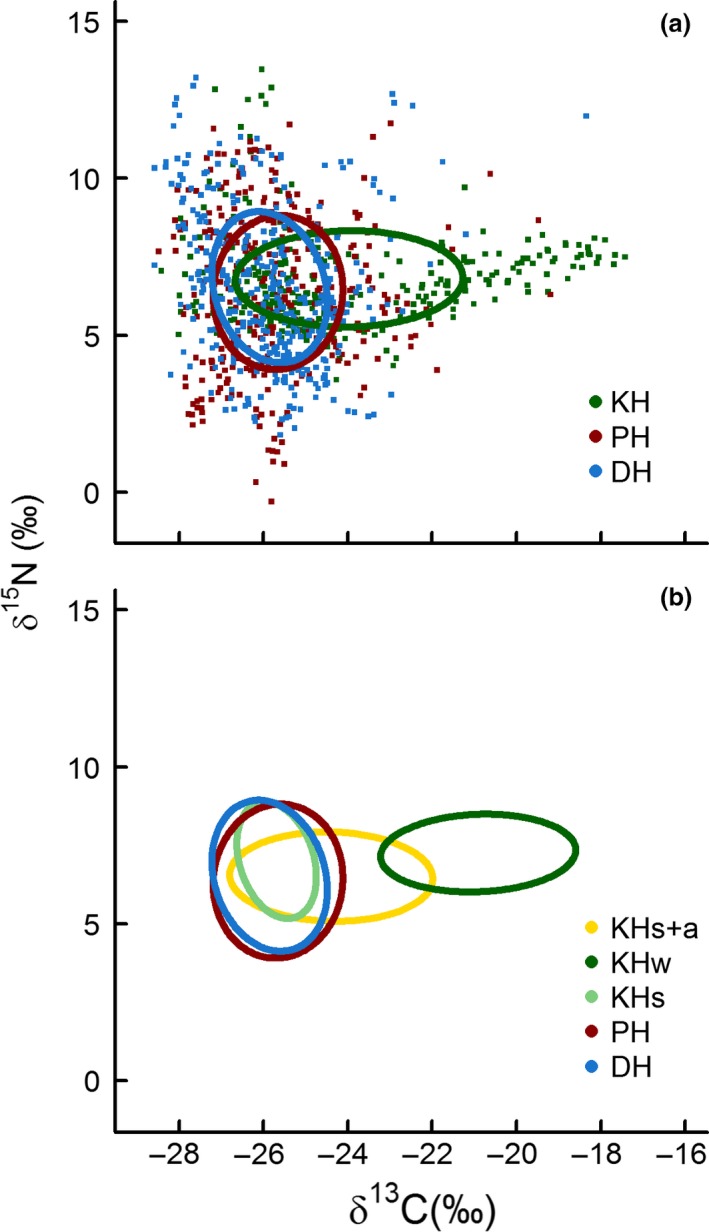
Bayesian standard ellipses (SEA_B_) representing estimated core isotopic niche widths: (a) Annual niches of khulan (KH), Przewalski's horses (PH), and domestic horses (DH) and (b) seasonal niches of khulan in summer (KHs), winter (KHw) and spring + autumn (KHs + a), and annual niches of the two horse species.

On a species level, the sizes of the core isotopic dietary niches were similar (SEA_B_: khulan = 13·0‰^2^, Przewalski's horses = 11·8‰^2^, domestic horses = 10·1‰^2^), with only the niche of domestic horses being slightly, but significantly smaller (domestic horses – khulan: *P *<* *0·001, domestic horses – Przewalski's horses: *P *=* *0·022; Fig. 3a).

Khulan exhibited a wider isotopic dietary niche along the δ^13^C axis, whereas both horse species exhibited wider niches along the δ^15^N axis (Fig. 3a). The isotopic dietary niches overlapped almost completely (95%) between Przewalski's and domestic horses, but only partly between khulan and Przewalski's horses (57%) or khulan and domestic horses (45%; Fig. 3a).

The isotopic dietary niches occupied by khulan during summer and winter were completely separated, and the spring + autumn niche was located in between (Fig. 3b). The size of the winter niche was much larger than the summer niche (SEA_B_: winter = 8·8‰^2^, summer = 5·3‰^2^, *P *<* *0·001), although similar to the spring + autumn niche (10·7‰^2^, *P *=* *0·104). The dietary niches of Przewalski's and domestic horses did not differ with season.

The two horse species’ annual niches overlapped completely with the khulan summer niche, but only partly with the khulan spring + autumn niche (Przewalski's horse = 59·1% and domestic horse = 48·2%; Fig. 3b), and not at all with the khulan winter niche (Fig. 3b).

At the individual level, khulan showed only small variations in the sizes of the isotopic dietary niches (SEA_B_ = 12·0 ± 3·5‰^2^) and a high average pairwise overlap of 82·7 ± 15·2%. The variation in niche size among individual domestic horses (SEA_B_ = 8·7 ± 3·8‰^2^) was similar to khulan, but slightly smaller in Przewalski's horses (SEA_B_ = 7·2 ± 2·1 ‰^2^). However, pairwise overlap among individual niches in both horse species was much smaller than in khulan, averaging 49·5 ± 23·5% in domestic and 32·5 ± 17·6% in Przewalski's horses. Furthermore, in Przewalski's horses, only 9 of 15 possible pairs had any overlap at all (Fig S4.1, Appendix S4).

## Discussion

### The importance of grass in the diet

Przewalski's horses in Mongolia showed that even under the extreme conditions in the Gobi, and despite the seasonal presence of livestock, it is possible to maintain a year‐round grazing diet. The same is true for domestic horses, but their access to grass‐dominated habitats is facilitated by herding. Contrary to wild and domestic horses, khulan show a strong seasonality with grazing during the summer, pronounced mixed feeding during the non‐growing season in winter, and a switch from one mode to the other during spring and autumn. The higher use of shrubs by khulan in winter is well in line with observations by local people, the literature (Bannikov 1981; Xu *et al*. 2012) and recent GPS data which showed higher khulan presence in *H. ammodendron* communities in winter (Appendix S5). However, the total lack of seasonality in the diet of horses is not consistent with observations of Przewalski's and domestic horses seeking shelter in *H. ammodendron* stands during winter (O. Ganbaatar, personal communications). We thus caution against the assumption that the presence in a certain plant community type automatically translates to diet or vice versa. In *H. ammodendron* stands, individual shrubs are often spaced quite far apart and forbs and grasses are found in the gaps. Furthermore, Przewalski's horses may switch between shrub‐dominated areas for shelter and adjacent more grass‐dominated patches for food which is not immediately apparent from visual observations.

The presence of herder camps has been shown to negatively affect the occurrence of khulan and other wild ungulates elsewhere in Mongolia (Olson *et al*. 2011; Young *et al*. 2011). Khulan in the Dzungarian Gobi are very wary of humans, and carcass remains suggest moderate levels of poaching. Khulan are also reportedly chased away from the vicinity of winter camps as herders consider them pasture competitors. Przewalski's horses, however, are still rare and have become a highly valued ‘flagship’ species of Mongolian nature (Kaczensky *et al*. 2006a; Kaczensky 2007). Many of the reintroduced Przewalski's horses in the SPA are still fairly relaxed about people due to their captive origin and lack of negative experiences. As a consequence, the displacement effect of humans on Przewalski's horses is likely less than on khulan. In fact, observations of Przewalski's horses grazing next to domestic horses in winter are not uncommon (O. Ganbaatar, personal observations). Thus, khulan switching to a mixed diet in winter may be the result of a broader dietary niche, different physiological needs (see Physiological adaptations further down**)**, a lower tolerance for human disturbance or any combination of these factors.

### Isotopic dietary niches and competition potential

The isotopic dietary niches of the two horse species were almost identical and remained constant throughout the year. Similar findings were made in central Mongolia based on micro‐histological analysis of faeces (Sietses *et al*. 2009). Due to the pronounced seasonality in khulan, the winter isotopic dietary niche did not overlap with the summer niche or the year‐round niches of Przewalski's and domestic horses. The winter isotopic dietary niche of khulan was much broader than the summer niche, which is again in line with findings of micro‐histological analysis of khulan faeces from adjacent areas of northern China (Xu *et al*. 2012).

In our study area, the potential for competition among large herbivores in summer is probably low because livestock, which currently constitutes the vast majority of ungulate grazers, leave the SPA in summer (Kaczensky *et al*. 2006a). Summer forage is relatively plentiful in the Dzungarian Gobi because most rain falls in June and July. The bottleneck is the non‐replenishable winter forage at times when low ambient temperatures require the highest energy expenditure to produce enough body heat to cope with the extreme low ambient winter temperatures (Kerven 2004; Arnold, Ruf & Kuntz 2006). Consequently, pasture competition among wild and domestic ungulates is most likely to occur in late winter when body condition is lowest and time since cessation of plant growth is longest. The results of our study, combined with studies from elsewhere in the region, currently suggest a high competition potential between the two horse species, but only a limited competition potential between both horse species and khulan.

Khulan may compete more strongly with other livestock known to include browse in their diet like camels and goats (Zhao *et al*. 2006; Animut & Goetsch 2008). The latter have become the dominant livestock in the Gobi and tend to be kept in mixed flocks with sheep, enhancing the herds potential to exploit mixed grass‐shrub pastures and thus constituting powerful pasture competitors for a wide range of wild herbivores if resources are limited (Animut & Goetsch 2008; Berger, Buuveibaatar & Mishra 2013). Correlative evidence suggests that khulan may not be able to tolerate high stocking rates (Kaczensky *et al*. 2011b), although we are yet to understand if this threshold reflects a resource limitation or rather is a threshold for overall ‘human impact’ (i.e. including disturbance and illegal killings).

### Individual variation

Contrary to both horse species, khulan were highly synchronized in their isotopic profile and variation in the isotopic dietary niches among individual khulan was much smaller than among individual Przewalski's and domestic horses. This came as a surprise because Przewalski's and domestic horses are known to live in stable, highly coordinated social groups (Souris *et al*. 2007), whereas khulan live in fission–fusion groups with little movement coordination (Rubenstein *et al*. 2015). However, in Przewalski's and domestic horses, we foremost sampled across and not within social groups and the ranges of the different groups did not, or only partly, overlap. Consequently, different individuals had access to different micro‐ and macrohabitats. Khulan, however, are highly mobile habitat generalists, and in our study area, all individuals range pretty much across the total range of the population (Kaczensky *et al*. 2008, 2011b). Consequently, individual khulan are likely to ‘average’ the isotopic values of their preferred diet over a large area, whereas the more spatially confined Przewalski's and domestic horse groups reflect local differences in the isotopic values of their diet.

### Physiological adaptations

Desert‐adapted ungulates have evolved physiological adaptations that reduce the amount of water lost through cutaneous and pulmonary evaporation, faeces and urine (Cain *et al*. 2005). Dehydrated ungulates are known to conserve water by reducing total urine volume and increasing concentration (Cain *et al*. 2005 and references therein), resulting in increased body tissue δ^15^N values (Ambrose & DeNiro 1986; Sealy *et al*. 1987; Sponheimer *et al*. 2003). Higher δ^15^N values in khulan suggest that they need to drink less or can tolerate longer inter‐drink intervals than the two horse species, supporting inferences from previous studies, including the observation that khulan exploit pastures further away from water than Przewalski's horses (Scheibe *et al*. 1998; Kaczensky *et al*. 2008). However, conserving water also means increased loss of N due to a disproportionate excretion of ^15^N‐depleted urea into the urine. In winter, the protein content of dry grasses is lowest (Sealy *et al*. 1987), whereas it is generally believed to remain rather constant in C_4_ shrubs. Hence, an increased need to replenish N in winter may be an additional driver for the increased consumption of C_4_ shrubs by khulan.

Starvation is preceded by metabolism of fat and as a last resort muscle tissue, and we had expected to see increased δ^15^N values in winter, especially in Przewalski's horses which all had succumbed to the 2009/2010 dzud winter (Kaczensky *et al*. 2011a). The lack of consistently increased δ^15^N values in late winter suggests that all three species are able to accumulate enough fat reserves to last them through the winter. Those individuals which died during the dzud winter probably did not die of starvation (a long‐term process), but rather froze to death as a consequence of imminent fodder lack which greatly reduces heat production from the large digestive tract of herbivores (Kerven 2004).

For H, we found a clear and consistent seasonal pattern in all species, with high δ^2^H values during summer and low δ^2^H values during winter matching the extreme seasonality of δ^2^H in local precipitation (see Burnik Šturm *et al*. 2015). The highest amplitude of the δ^2^H pattern seen in Przewalski's horses is likely a result of their dependence on a limited set of water sources. The lack of a strong seasonal trend in δ^2^H for domestic horses can be attributed to their seasonal migrations to high altitude pastures in summer, where δ^2^H values of precipitation are lower (Meier‐Augenstein 2010), and hence, the seasonal difference between summer and winter precipitation becomes less pronounced. Due to a general lack of understanding of the behaviour of δ^2^H in animals, we are currently unable to explain the significantly higher δ^2^H values in khulan.

## Conservation implications

The similarity of the isotopic patterns and isotopic dietary niches points towards a high competition potential between the closely related Przewalski's and domestic horses, whereas a different isotopic pattern in khulan coupled with their high mobility and their ability to exploit pastures further away from water suggests a lower competition potential between khulan and the two horses. Given the expanding Przewalski's horse population and the precarious state of khulan outside of Mongolia, protected area management should aim to severely restrict grazing of domestic horses and reduce, or at least prevent, any increase in grazing of other livestock. Furthermore, rehabilitating currently broken livestock wells or providing additional artificial water sources should be avoided as it will likely increase the potential for competition between khulan and livestock (Kaczensky *et al*. 2006b). In the light of increasing livestock numbers throughout Mongolia and Central Asia, we see a great need for future studies to better understand pasture competition between the full range of wild and domestic ungulates.

## Data accessibility

The raw tail hair isotope data can be accessed from Dryad Digital Repository http://dx.doi.org/10.5061/dryad.16r15 (Burnik Šturm *et al*. 2016).

## Supporting information


**Appendix S1.** Supplementary information on livestock in Mongolia, the Dzungarian Gobi, and Central Asia.
**Appendix S2.** Plant sampling and stable isotope values of plants in Great Gobi B SPA.
**Appendix S3.** Stable isotope analysis methods and isotope values in hair.
**Appendix S4**. Estimation of isotopic niche widths.
**Appendix S5**. GPS locations of khulan in Great Gobi B SPA.
