# Supplementary material for: Sequential stable isotope analysis reveals differences in dietary history of three sympatric equid species in the Mongolian Gobi
Source: J Appl Ecol. 2016 Nov 17;54(4):1110–9. doi: 10.1111/1365-2664.12825 (PMC5510718; doi:10.1111/1365-2664.12825)
Supplement: Supplementary file 1 — Appendix S1. Supplementary information on livestock in Mongolia, the Dzungarian Gobi, and Central Asia. Appendix S2. Plant sampling and stable isotope values of plants in Great Gobi B SPA. Appendix S3. Stable isotope analysis methods and isotope values in hair. Appendix S4. Estimation of isotopic niche widths. Appendix S5. GPS locations of khulan in Great Gobi B SPA. [file JPE-54-1110-s001.docx]

**Appendix S1:** Supplementary information on livestock in Mongolia, the Dzungarian Gobi, and Central Asia.

**Fig S1.1.** Livestock trend and composition in Mongolia.


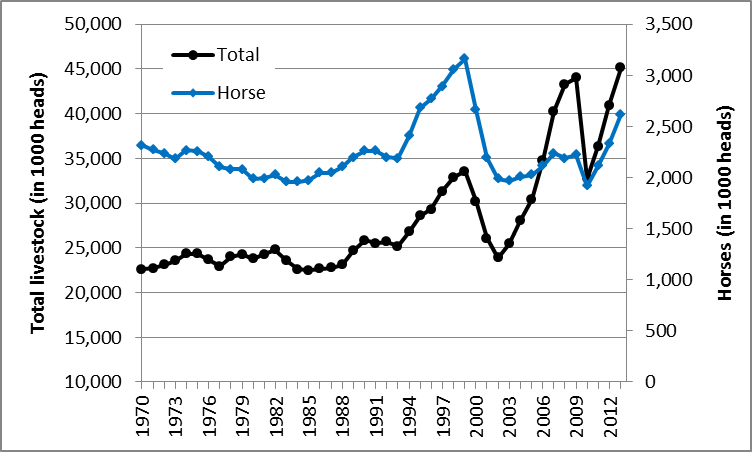


recent "dzud" events

1. Livestock trend in Mongolia 1970-2013.


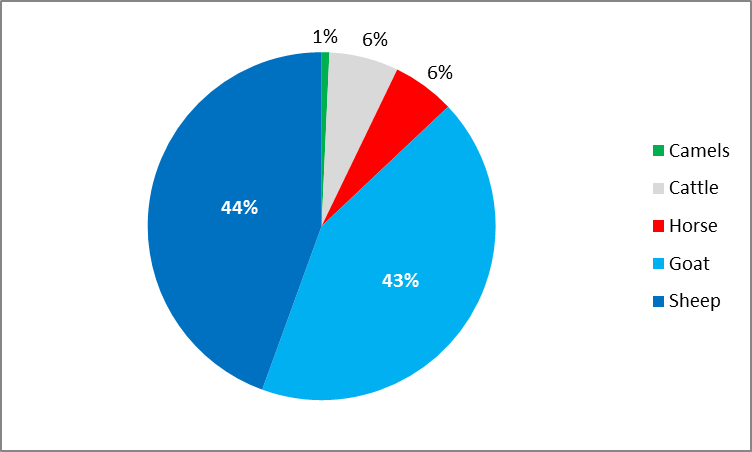


1. Livestock composition in Mongolia 2013.

**Fig S1.2.** Livestock trend and composition in the Dzungarian Gobi in SW Mongolia.


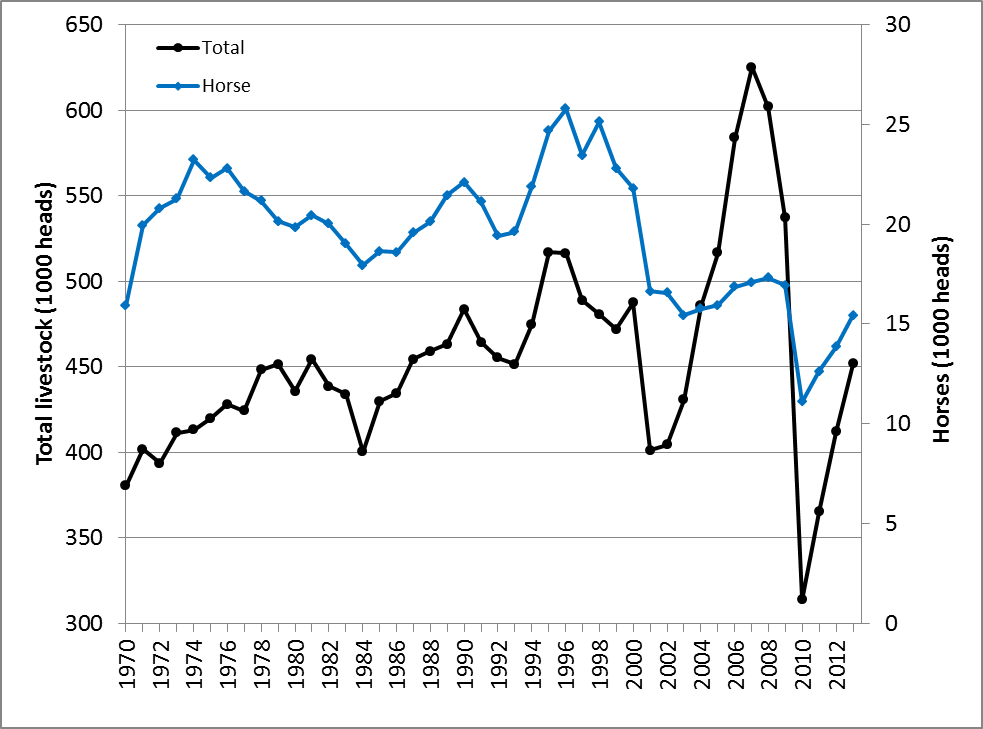


recent "dzud" events

1. Livestock trend in the four districts (Bugat, Tonkhil, Khovd, Uench) containing the Great Gobi B SPA 1970-2013.


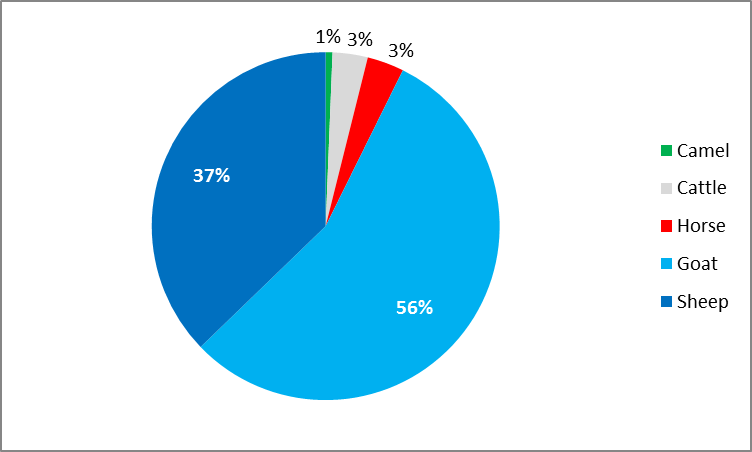


1. Livestock composition in the four districts (Bugat, Tonkhil, Khovd, Uench) containing the Great Gobi B SPA 2013.

**Fig S1.3.** Representation of *Stipa* (C_4_ grass) and *Haloxylon* (C_3_ shrub) dominated plant communities in a 5 km grazing buffer around winter camps in and adjacent to Great Gobi B SPA in SW Mongolia.


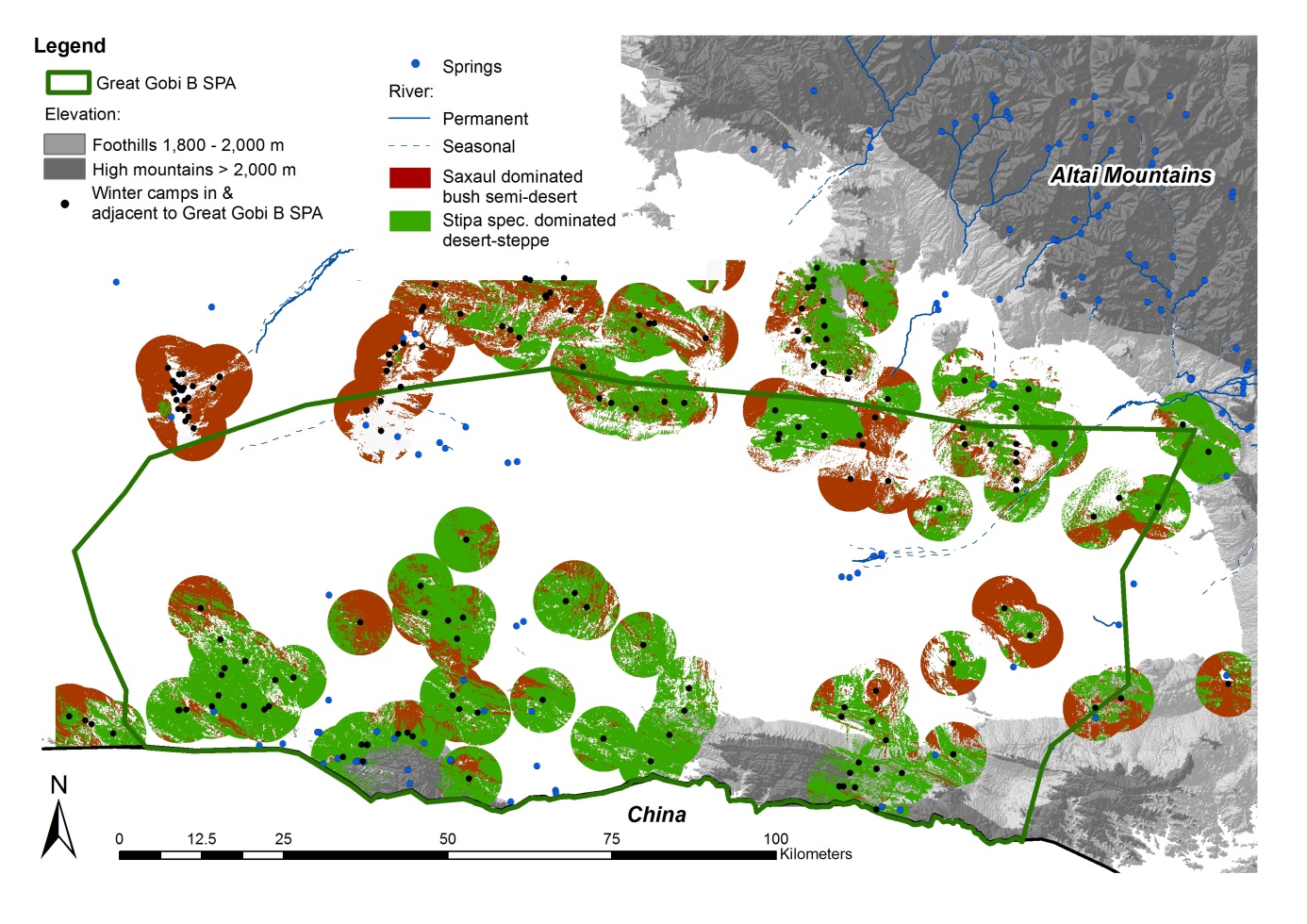
In Great Gobi B SPA most winter camps are in or near grass dominated plant communities (also see Figure 1). The average grazing range around a winter camp is about 5 km and the plant community composition within a 5 km buffer around all winter camps in the SPA and the immediate surrounding consists of 46% grassdominated *Stipa* and 28% *Haloxylon* dominated plant communities.

Livestock numbers within Great Gobi B SPA seem to largely follow the dynamics in the four districts which contain the SPA. Composition and trend in these districts is largely in line with the overall national trend, but also shows the higher importance of goats in the Gobi and the higher regional impact of the 2009/10 dzud winter (see Kaczensky *et al.* 2011).

**Fig S1.4.** Trend in sheep and goat numbers in Mongolia and Central Asia.


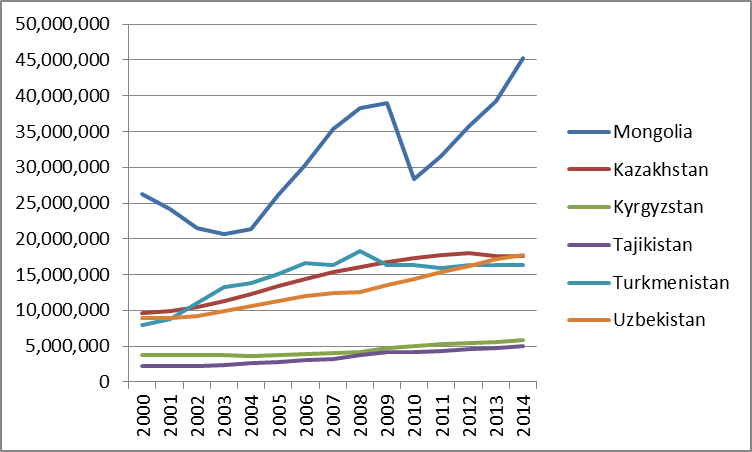


**Literature cited:**

Food and Agriculture Organization of the United Nations (FAO) – Statistics division, <http://faostat3.fao.org/download/Q/QA/E>

Kaczensky, P., Ganbataar, O., Altansukh, N., Enkhsaikhan, N., Stauffer, C. & Walzer, C. (2011) The danger of having all your eggs in one basket-winter crash of the re-introduced Przewalski’s horses in the Mongolian Gobi. *PloS One*, **6**, e28057.

Mongolian Statistical Information Service, <http://www.1212.mn/en/contents/stats/contents_stat_fld_tree_html.jsp>

**APPENDIX S2**: Plant sampling and stable isotope VALUES of plants IN GRET GOBI B SPA.

In 2012 we collected samples from 13 different species of C_3_ plants: *Stipa* sp., *Achnatherum splendens*, *Agropyron* sp., *Ajania* sp., *Allium* sp., *Artemisia* sp., *Caragana leucophloea*, *Elymus* sp., *Eurotia ceratoides*, *Festuca* sp., *Reaumuria soongorica*, *Zygophyllum* sp., *Phragmites australis,* and mixed grass samples and two species of C_4_ plants (*Haloxylon ammondendron* and *Anabasis brevifolia*). *Haloxylon* species have a very unusual photosynthetic apparatus where the cotlyledons follow the C_3_ photosynthetic pathway but the shoots follow a C_4_ pathway (Pyankov et al. 1999). Succulent CAM plants were not collected as they are very rare in Central Asia (Oyungerel et al. 2004, Lee et al. 2005). Samples were primarily collected at 24 evenly spaced grid points (20 km north-south and 40 km east-west, Fig. S2).

In 2013, we collected *Stipa* sp. and *Artemisia* sp. at 100 meter steps between 1400 and 2800 m above sea level to check for the potential effect of altitude and *Stipa* sp. and *Haloxylon* along the east–west gradient at 10 km steps to check for the potential effect of longitude on their isotope composition (Fig. S2). Sampling sites represent a range of different micro habitats, such as riparian zones, rocky and sandy plains, depressions, hills and mountain slopes.

In total we collected and analyzed 240 plant samples (2012, 2013). Each plant sample comprised of small parts from 5–10 individual plants of the same species. Plant samples were placed in individually labelled (species, date, GPS coordinates of sample location) paper envelope and air dried at the Takhin Tal field station.


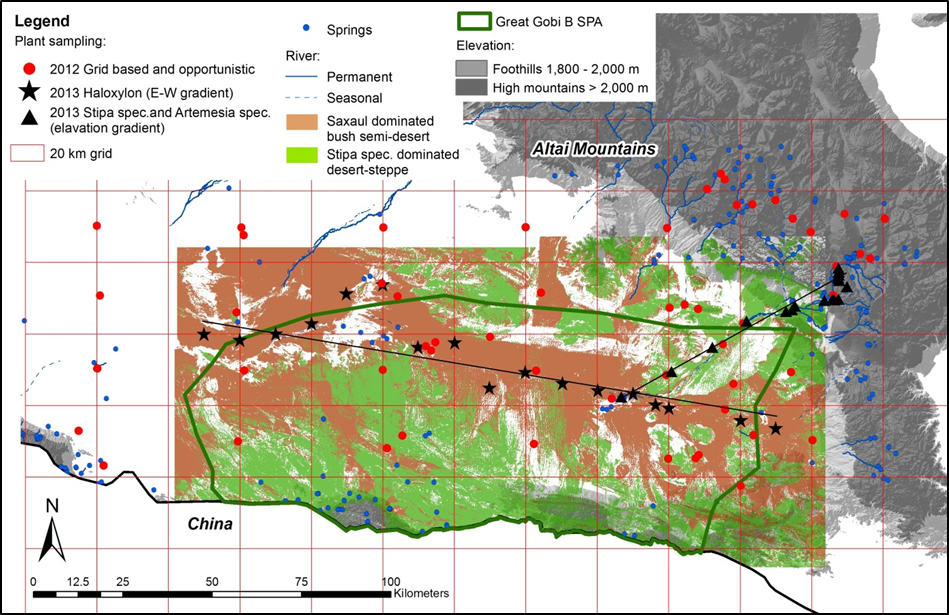


Fig S2. Plant sampling locations relative to *Stipa* (grass) and *Haloxylon* (shrub) dominated plant communities in Great Gobi B SPA, SW Mongolia.

Table S2. Stable isotope values (*δ*^13^C, *δ*^15^N) of plant samples by group and species from Great Gobi B SPA, SW Mongolia.

**Literature cited:**

Lee, X., Feng, Z., Guo, L. Wang, L., Jin, L. Huang, Y., Chopping, M., Huang, D., Jiang, W., Jiang, Q. & Cheng, H. (2005) Carbon isotope of bulk organic matter: A proxy for precipitation in the arid and semiarid central East Asia. *Global Biogeochemical Cycles*, **19**, GB4010, doi:10.1029/2004GB002303.

Oyungerel, S., Tsendeekhuu, T. & Tserenkhand, G. (2004) A Study to Detect CAM Plants in Mongolia. *Mongolian Journal of Biological Sciences*, **2**, 29–37.

Pyankov, V., Black, C.J., Artyusheva, E.G., Voznesenskaya, E.V., Ku, M.S.B. & Edwards, G.E. (1999) Features of photosynthesis in Haloxylon species of Chenopodiaceae that are dominant plants in Central Asian deserts. *Plant and Cell Physiology*, **40**, 125–134.

**Appendix S3.** Stable isotope analysis methods and isotope values in

hair.

Isotope analyses were conducted at the stable isotope facility at the Leibniz Institute for Zoo and Wildlife Research (IZW), Berlin.

Stable carbon and nitrogen isotope ratios were measured using an elemental analyzer (Flash EA, ThermoFisher Scientific, Bremen, Germany) connected in continuous mode to a stable isotope ratio mass spectrometer (Delta V-Advantage, ThermoFisher Scientific, Bremen, Germany). Sample isotope ratios were compared to international standards (V-PDB and air N) and expressed as *δ*^13^C and *δ*^15^N values in part per mille deviation from standard values.

For measuring stable isotope ratios of non-exchangeable hydrogen, samples were dried in a drying oven over 24 hours at 50°C and then placed into silver capsules (IVA Analysetechnik E.K., Meerbusch, Germany). Loaded capsules were then placed in a Zero Blank autosampler (Costech Analytical Technologies Inc. Italy) where they were flushed for at least an hour with chemically pure helium before dropping into the elemental analyzer (HT Elementaranalysator HEKAtech GmbH, Wegberg, Germany). The EA was connected via an interace (Finnigan Conflo III, ThermoFisher Scientific Bremen, Germany) to the stable isotope ratio mass spectrometer (Delta V advantage, ThermoFisher Scientific, Bremen, Germany).

To overcome the problem of uncontrolled hydrogen isotopic exchange between keratin and isotopically variable ambient moisture in the laboratory, we used the comparative equilibration method described by Wassenaar and Hobson (2003). Samples were analyzed together with previously calibrated in-house keratin hydrogen isotope reference materials: SWE-SHE (-167.9±1‰, sheep wool), ESP-SHE (-108.3±1‰, sheep wool), and AFR-GOA (-66.3±0.9‰, goat wool). Details on the preparation of the in-house keratin standards are described in Popa-Lisseanu *et al.* (2012). Stable hydrogen isotope ratios are expressed as delta value in per mille deviation from the international standard V-SMOW. Precision of the measurements was always better than 0.1 ‰ for *δ*^13^C and *δ*^15^N, and 1.0 ‰ for non-exchangeable *δ*^2^H values based on the repeated analysis of the laboratory standards, calibrated with the international standards.

**Literature cited:**

Wassenaar, L.I. & Hobson, K.A. (2003) Comparative equilibration and online technique for determination of non-exchangeable hydrogen of keratins for animal migration studies. *Isotopes in Environmental and Health Studies*, **39**, 211–217.

Popa-Lisseanu, A.G., Sörgel, K., Luckner, A., Wassenaar, L.I., Ibáñez, C., Kramer-Schadt, S., Ciechanowski, M., Görföl, T., Niermann, I., Beuneux, G., Mysłajek, R.W., Juste, J., Fonderflick, J., Kelm, D.H. & Voigt, C.C. (2012) A triple-isotope approach to predict the breeding origins of European bats. *PloS ONE*, **7**, e30388.

**Table S3.1.** Additional details on animals sampled.

**Khulan**

Khulan in Great Gobi B SPA don’t seem to form stable groups (Kaczensky et al. 2008) and we captured animals from a wide variety of possible group sizes. Animals were captured for a project aiming at understanding movement patterns and drivers of khulan movements and thus were equipped and subsequently followed by telemetry for up to 12 months. Although only a limited number of collars provided data over an entire year, GPS locations clearly showed that none of the collared khulan moved together for any longer periods of time (more than a few days at most; Kaczensky unpubl. data).

**Przewalski’s horses**

Przewalski’s horses live in stable harem groups, which are highly coordinated (Souris et al. 2007, Kaczensky et al. 2008, Boyd et al. 2016) and hence individuals of the same group can be expected to show similar isotope profiles. To confirm this assumption we sampled two individuals from the same group (Shijee group; which indeed had almost identical isotope profiles), but otherwise aimed to include known individuals from different groups and those with the longest tail hair. All animals sampled had succumbed to the 2009/2010 dzud winter and we selected those individuals which could be unequivocally identified by the rangers based on body shape and color markings.

**Domestic horses**

Domestic horses were opportunistically sampled at three different locations and after interviews confirmed that herders spend fall to spring in or along the fringes of the Great Gobi B Strictly Protected Area. Four of the six horses were sampled in summer at or near the grass dominated alpine pasture summer camps where C_4_ shrubs were absent, while two were sampled in late winter 2010 after the extreme 2009/10 dzud winter and in areas where they had access to C_4_ shrubs. Domestic horses move largely unguarded and often roam many tens of kilometers away from their owners gers (Kaczensky et al. 2006), but are moved with the rest of the livestock when camp locations are changed.

**Literature cited:**

Boyd, L., Scorolli, A., Nowzari, H., Bouskila, A. (2016) Social organization of wild equids. *Wild Equids - Ecology, Management, and Conservation* (eds J.I.Ransom & P. Kaczensky), pp. 7-22 Johns Hopkins University Press, Baltimore, USA.

Kaczensky, P., Enkhsaihan, N., Ganbaatar, O., Walzer, C. (2006) Identification of herder-wild equid conflicts in the Great Gobi B Strictly Protected Area in SW Mongolia. *Exploration into the Biological Resources of Mongolia,* **10**, 99-116.

Kaczensky, P., Ganbaatar, O., von Wehrden, H., Walzer, C. (2008) Resource selection by sympatric wild equids in the Mongolian Gobi. *Journal of Applied Ecology,* **45**, 1762–1769.

Souris, A.C., Kaczensky, P., Julliard, R., Walzer, C. (2007) Time budget-, behavioral synchrony- and body score development of a newly released Przewalski's horse group Equus ferus przewalskii, in the Great Gobi B Strictly Protected Area in SW Mongolia. Applied Animal Behaviour Science, **107**, 307-321.

Table S3.2. Mean stable isotope values in individual tails of three equid species in the Dzungarian Gobi, SW Mongolia.

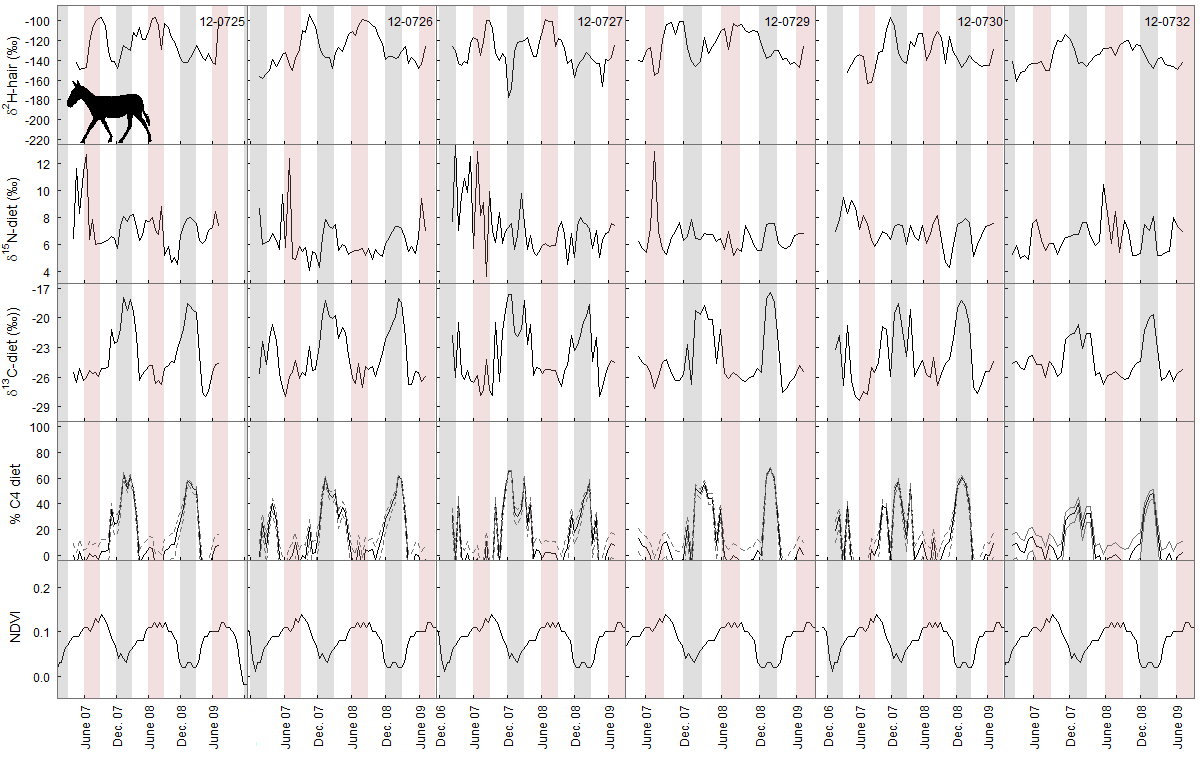


Fig S3.1. Sequential, temporal explicit, individual khulan *δ*^2^H_hair_, *δ*^15^N_diet_, *δ*^13^C_diet_, and % C_4_ diet profiles and time-matched 16-day NDVI values. The fraction of C_4_ biomass in the diets takes into account the isotopic variability of the C_3_ and C_4_ end members used in the mixing model (mean *δ*^13^C ± 1σ, depicted with solid and dashed line, respectively). Pink and gray stripes depict the summer and winter, respectively.


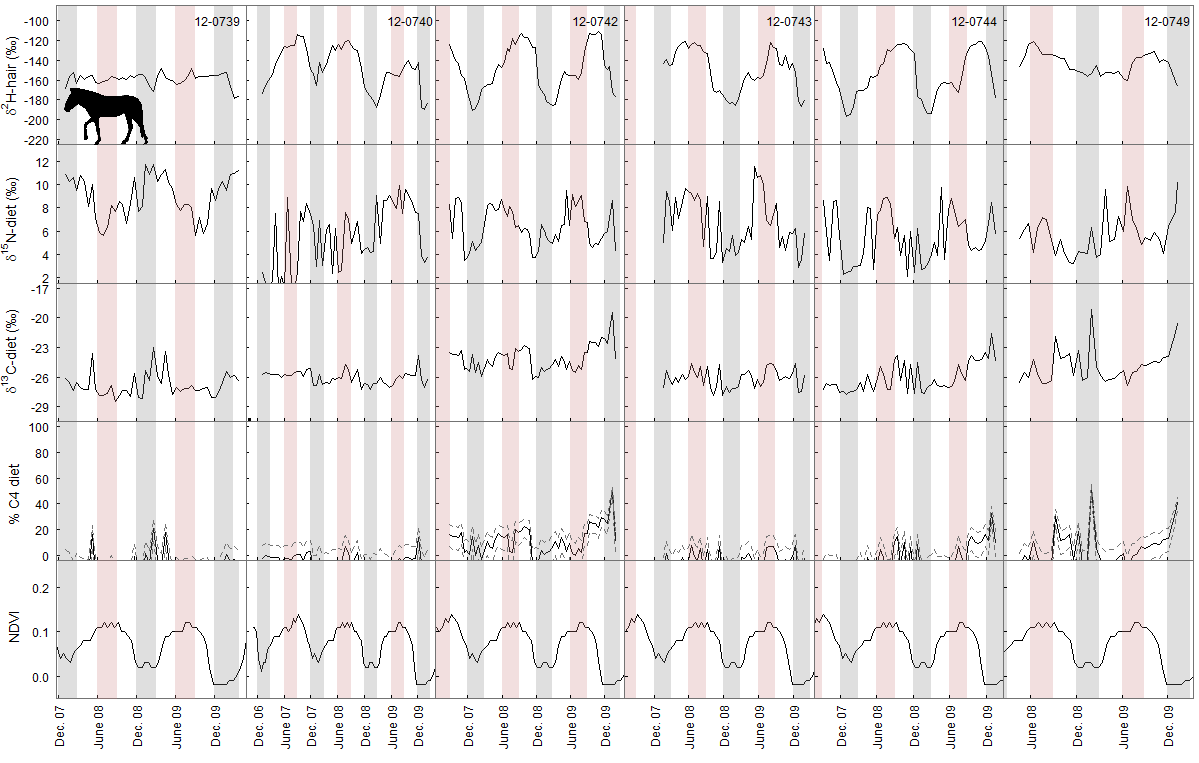


Fig S3.2. Sequential, temporal explicit, individual Przewalski’s horse *δ*^2^H_hair_,* *^15^N_diet_, *δ*^13^C_diet_, and % C_4_ diet profiles and time-matched 16-day NDVI values. The fraction of C_4_ biomass in the diets takes into account the isotopic variability of the C_3_ and C_4_ end members used in the mixing model (mean *δ*^13^C ± 1σ, depicted with solid and dashed line, respectively). Pink and gray stripes depict the summer and winter, respectively.


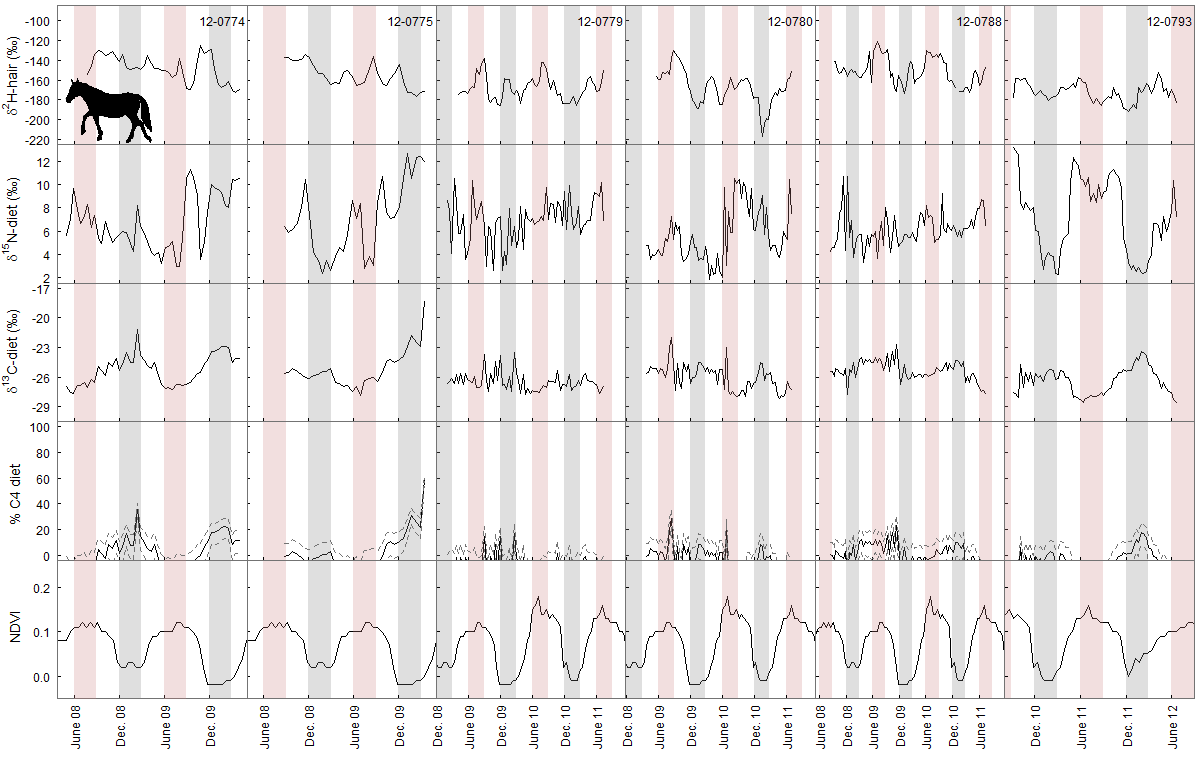


Fig S3.3. Sequential, temporal explicit, individual domestic horse *δ*^2^H_hair_, *δ*^15^N_diet_, *δ*^13^C_diet_, and % C_4_ diet profiles and time-matched 16-day NDVI values. The fraction of C_4_ biomass in the diets takes into account the isotopic variability of the C_3_ and C_4_ end members used in the mixing model (mean *δ*^13^C ± 1σ, depicted with solid and dashed line, respectively). Pink and gray stripes depict the summer and winter, respectively.

**APPENDIX S4**. Estimation of isotopic niche widths

We estimated the isotopic niche width and overlap using a Bayesian approach based on multivariate, ellipse–based metrics (Jackson et al. 2011). This method is robust for comparison between small sample sizes, as it identifies differences in the niche width of “typical” members of the population, but excludes “outlier” individuals. It provides a single measure on a continuous axis that is common to all species and hence enables comparison between populations and species on a single scale (Bearhop et al. 2004).

The analysis was performed using SIBER (Stable Isotope Bayesian Ellipses) implemented in the R package SIAR (Parnell et al. 2010) to generate standard ellipse areas (SEA_B_): a bivariate equivalent to standard deviation and a corrected measure for small sample sizes (SEA_C_). The area within an ellipse was defined by a subsample containing 40 % of the bivariate data (*δ*^15^N and *δ*^13^C) and thus represents the core niche area for a species or individual (Jackson et al. 2011).

Statistical significance of differences in SEA_C_ between sample groups was based on the proportional outcome of 10,000 repeat measures. This approach was used to estimate the levels of niche overlap (0 – 100 %, suggesting no to complete overlap, respectively) between species as well as between individuals of the same species to check for the degree of individual specialization. We also compared the overlaps in summer and winter dietary niches of khulan with those of the two horse species.

We converted the stable isotope ratios of equid hair (*δ*^15^N_hair_, *δ*^13^C_hair_) to diet coordinates (*δ*^15^N_diet_, *δ* ^13^C_diet_), thus the generated ellipses are representative of the respective isotopic dietary niche in the isotopic space of the Mongolian Great Gobi B SPA which is defined by *δ*^13^C and *δ*^15^N of local plants.


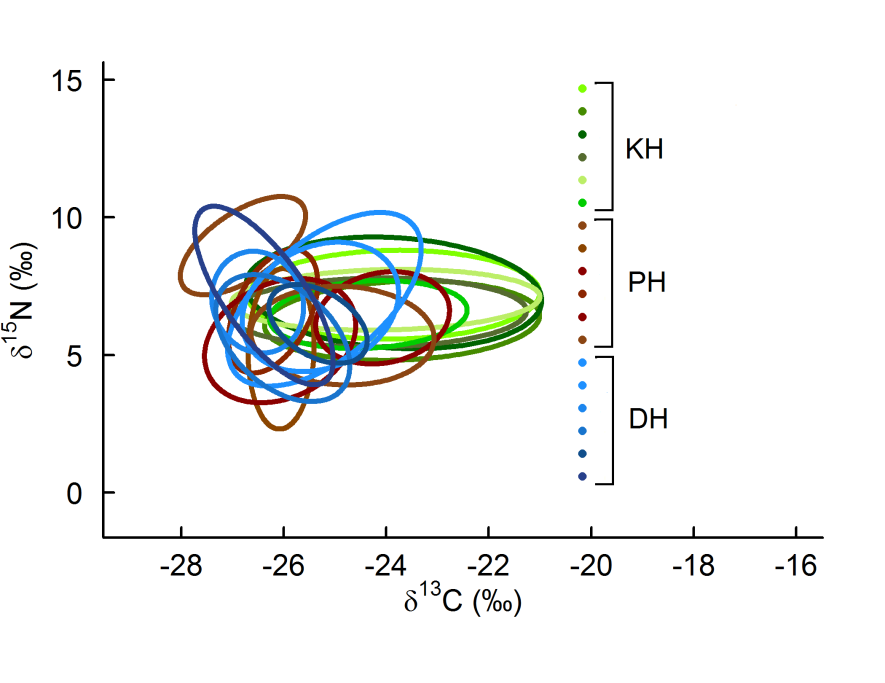


Fig S4.1. Bayesian standard ellipses (SEA_B_) representing estimated isotopic niche widths of each of the six individuals in khulan (KH), Przewalski’s horse (PH) and domestic horse (DH).

**Literature cited:**

Bearhop, S., Adams, C., Waldron, S., Fuller, R. & Macleod, H. (2004) Determining trophic niche width: a novel approach using stable isotope analysis. *Journal of Animal Ecology,* **73**, 1007–1012.

Jackson, A.L., Inger, R., Parnell, A.C. & Bearhop, S. (2011) Comparing isotopic niche widths among and within communities: SIBER - Stable Isotope Bayesian Ellipses in R. *The Journal of Animal Ecology,* **80**, 595–602.

Parnell, A.C., Inger, R., Bearhop, S. & Jackson, A.L. (2010) Source partitioning using stable isotopes: coping with too much variation. *PloS ONE*, **5**, e9672.

**APPENDIX S5**: GPS locations of khulan in Great Gobi B SPA.

**Table S5.** Monitoring period, number of GPS locations, and proportion in *Haloxylon ammodendrum* dominated communities by month for six khulan in the Dzungarian Gobi, SW Mongolia. GPS locations were obtained at 15-min intervals.

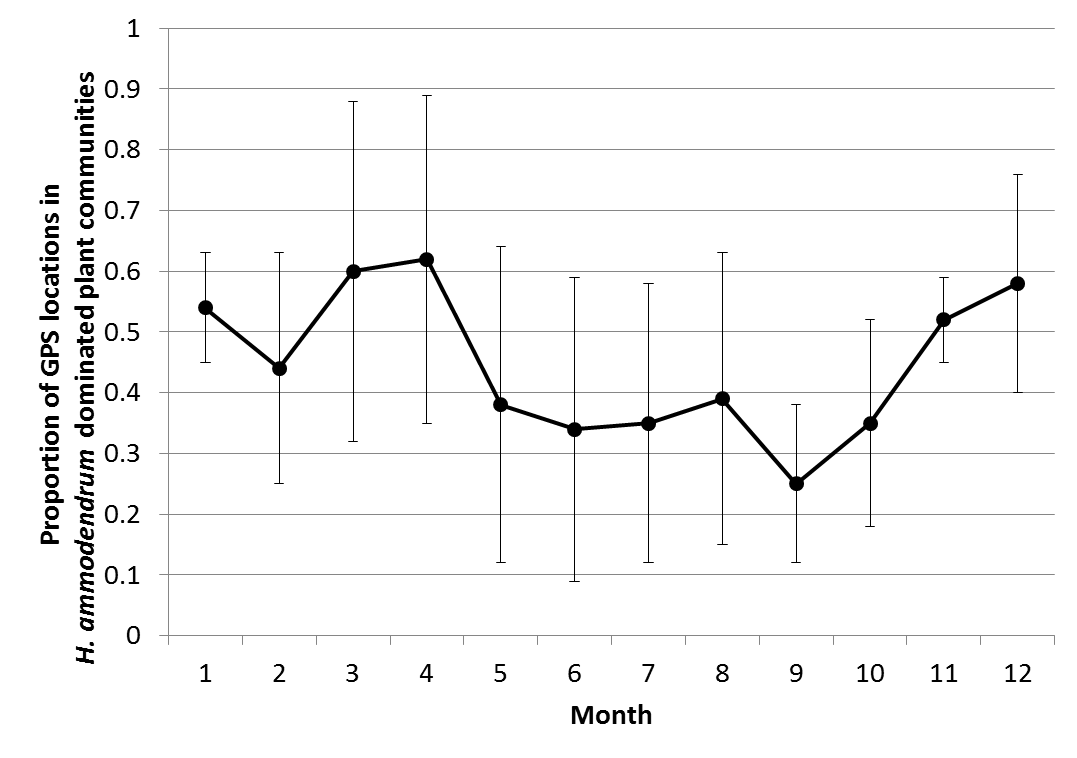


**Fig S5.** Proportion of GPS locations of six khulan in *Haloxylon ammodendrum* dominated plant communities in the Dzungarian Gobi, SW Mongolia.
